# A102 EUS-GUIDED GASTROENTEROSTOMY IS COST-EFFECTIVE COMPARED TO SURGICAL GASTROJEJUNOSTOMY AND ENTERAL STENTING FOR PALLIATION OF MALIGNANT GASTRIC OUTLET OBSTRUCTION

**DOI:** 10.1093/jcag/gwad061.102

**Published:** 2024-02-14

**Authors:** C Miller, Y Rostamianmoghaddam, A Khakban, A Barkun, Y Chen

**Affiliations:** McGill University, Montreal, QC, Canada; McGill University, Montreal, QC, Canada; The University of British Columbia, Vancouver, BC, Canada; McGill University, Montreal, QC, Canada; McGill University, Montreal, QC, Canada

## Abstract

**Background:**

Endoscopic ultrasound-guided gastroenterostomy (EUS-GE) is an emerging modality for the treatment of malignant gastric outlet obstruction (GOO), which combines the benefits of the traditional treatments by providing a robust bypass using a minimally invasive, endoscopic approach. The health economic aspects of this new treatment compared to the traditional treatment approaches are not known.

**Aims:**

To conduct a cost-consequence analysis to compare the costs and benefits of using EUS-GE, surgical gastrojejunostomy (SGJ) and enteral stenting (ES) for the management of malignant GOO.

**Methods:**

A decision analytical model, comprising a decision tree and a time-dependent state-transition model with three health states (no recurrent GOO, recurrent GOO, and death), was designed. The costs and benefits of the first month are included in the decision tree, after which, simulated patients enter the time-dependent state-transition model and the costs and consequences of each intervention are estimated for each monthly cycle until the end of a twelve-month time horizon. To address uncertainty around parameters, the expected values of costs and other outcomes were obtained through probabilistic analysis. The model parameters were assigned probability distributions and 10,000 Monte Carlo simulations were conducted using randomly sampled values drawn from these distributions. The outcomes were the estimated total cost and the probability of recurrent GOO in the follow-up time. We compared the incremental cost of avoiding recurrent GOO between these three strategies. A willingness-to-pay threshold of $50,000 to avoid recurrent GOO was established a priori.

**Results:**

The total estimated costs for EUS-GE, SGJ, and ES, were $82,575 (SD, $16,240), $149,731 (SD, $27,254), and $77,324 (SD, $14,529), respectively. SGJ (6.84% [SD, 1.34]) had a lower GOO recurrence rate compared to EUS-GE (8.70% [SD, 1.64] and ES (23.37% [SD, 3.82]; however, when comparing SGJ with EUS-GE and ES, the incremental cost to avoid GOO recurrence was $ 3,611,345 and $37,854, respectively. The cost of avoiding GOO recurrence using EUS-GE compared to ES was $35,774.

**Conclusions:**

EUS-GE has emerged as a cost-effective option in treating patients with malignant GOO. It is associated with a much lower cost per avoided recurrent GOO when compared to SGJ, and it is more effective in preventing recurrent GOO than ES at a cost that is well below the pre-set willingness-to-pay threshold of $50,000.

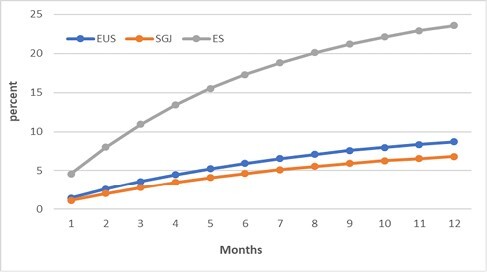

Cumulative rate of GOO recurrence for EUS-GE, SGJ and ES

**Funding Agencies:**

None

